# Predicting DNA binding protein-drug interactions based on network similarity

**DOI:** 10.1186/s12859-020-03664-6

**Published:** 2020-07-20

**Authors:** Wei Wang, Hehe Lv, Yuan Zhao

**Affiliations:** 1 Department of Computer Science and Technology, College of Computer and Information Engineering, Henan Normal University, Xinxiang, 453007 China; 2 Big Data Engineering Laboratory for Teaching Resources & assessment of Education Quality, Henan Province, Xinxiang, China

**Keywords:** DNA binding protein, Amino acid trimer, Cluster, Network

## Abstract

**Background:**

The study of DNA binding protein (DBP)-drug interactions can open a breakthrough for the treatment of genetic diseases and cancers. Currently, network-based methods are widely used for protein-drug interaction prediction, and many hidden relationships can be found through network analysis. We proposed a DCA (drug-cluster association) model for predicting DBP-drug interactions. The clusters are some similarities in the drug-binding site trimmers with their physicochemical properties. First, DBPs-drug binding sites are extracted from scPDB database. Second, each binding site is represented as a trimer which is obtained by sliding the window in the binding sites. Third, the trimers are clustered based on the physicochemical properties. Fourth, we build the network by generating the interaction matrix for representing the DCA network. Fifth, three link prediction methods are detected in the network. Finally, the common neighbor (CN) method is selected to predict drug-cluster associations in the DBP-drug network model.

**Result:**

This network shows that drugs tend to bind to positively charged sites and the binding process is more likely to occur inside the DBPs. The results of the link prediction indicate that the CN method has better prediction performance than the PA and JA methods. The DBP-drug network prediction model is generated by using the CN method which predicted more accurately drug-trimer interactions and DBP-drug interactions. Such as, we found that Erythromycin (ERY) can establish an interaction relationship with HTH-type transcriptional repressor, which is fitted well with silico DBP-drug prediction.

**Conclusion:**

The drug and protein bindings are local events. The binding of the drug-DBPs binding site represents this local binding event, which helps to understand the mechanism of DBP-drug interactions.

## Background

DNA-binding proteins (DBPs) play a vital role in cell life activities such as DNA replication and RNA transcription. The DBPs have gained wide attentions because of their essential functions in a variety of biological processes [1]. Therefore, the research on the relationship between DBPs and drugs can provide a new idea for the treatment of genetic diseases and cancer by bioinformatics method. As many studies have found, DNA-binding protein 43 (TDP-43) causes amyotrophic lateral sclerosis (ALS) and the increased presence of TDP-43 in the cytoplasm is a prominent histopathological feature of degenerating neurons in various neurodegenerative diseases [2]. BRD4, a DNA-binding protein, has been dedicated to the development of DNA-binding protein-related drugs since it has been shown to be effective in blocking cell proliferation in cancer and cytokine production in acute inflammation [3]. Zhao studied the resistance of lung cancer DNA-binding protein (SSDBP1) to cisplatin and determined that SSDBP1 promotes cell survival and cisplatin resistance in human lung cancer cell lines [4]. These studies have shown that DBPs play a crucial role in basic life activities. These predictive studies of DBPs are currently important research topics for the treatment of underlying diseases and drug development [5–7].

There are currently two basic theories based on targets and ligands: the ligands that interact with the same target share some similar structures, and drugs similar to these ligands may bind to the target; the targets that bind to the same ligand share certain similar characteristics, and proteins similar to these targets may bind to the ligand [8–11]. Most existing methods predict possible interactions on machine learning [12–15]. Although these methods have higher prediction accuracy, they are impossible to study the mechanism of action between proteins and drugs from the binding site. Though there are some researches on the DBPs, few attentions have been paid on the drugs interact with DBPs base on bioinformatics method. In this work, we applied DBP-drug interactions network to analyze the DBPs-drug binding site fragments.

With the development of biotechnology, the search for interactions between DBPs and drugs is an important part of genomic drug discovery. For now, many significant progresses in technical development have been predicted protein-drug interactions on biological network. Cheng proposed a network-based inference (NBI) approach that used only the binary similarity of the target’s topological network to infer new targets for known drugs [16]. Lu used the similarity indices to predict protein-drug interactions and had achieved good predictive results in the MATADOR database [17]. Zong proposed a drug-target prediction method based on similarity, which proved that it can provide a promising solution for drug and target prediction based on the similarity of heterogeneous networks [18]. Alaimo proposed a recommendation technique based on binary network projection to realize resource transfer within the network [19]. Liu proposed a protein-drug network based label propagation algorithm and predicted new drugs that have extended lifespan to nematodes [20]. Emig proposed a web-based approach to predicting targets for specific diseases, which can retarget specific disease targets for a given disease [21]. In these methods, a large number of drug-protein interaction entries are used to build the network to ensure the accuracy of network predictions.

**Fig. 1:**
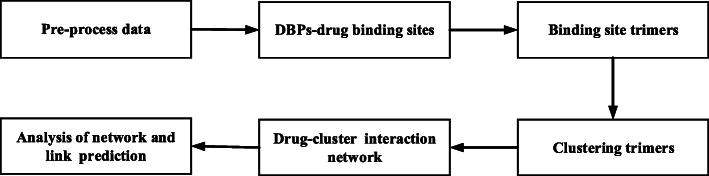
The whole workflow of our method

Although these methods gain good results in protein-drug interaction predictions, there are not deep studies on the intrinsic factors of protein-drug interactions. It is also impossible to explain the binding mechanism from internal factors. Therefore, we pay more attentions to the binding sites of DBPs, expecting to uncover the potential inherent factors in the DBP-drug interactions. In this article, based on the fact that the DBP-drug interactions are more local events, we used the trimers (local information) to describe the drug-binding sites. The specific process is shown in Fig. 1. Furthermore, we assume that the drug-binding sites of trimer interactions determine the DBP-drug interaction. We propose a drug-cluster association model to predict DBP-drug interactions and to clarify internal binding mechanisms.

## Results

### Investigation on the interaction data

**Fig. 2:**
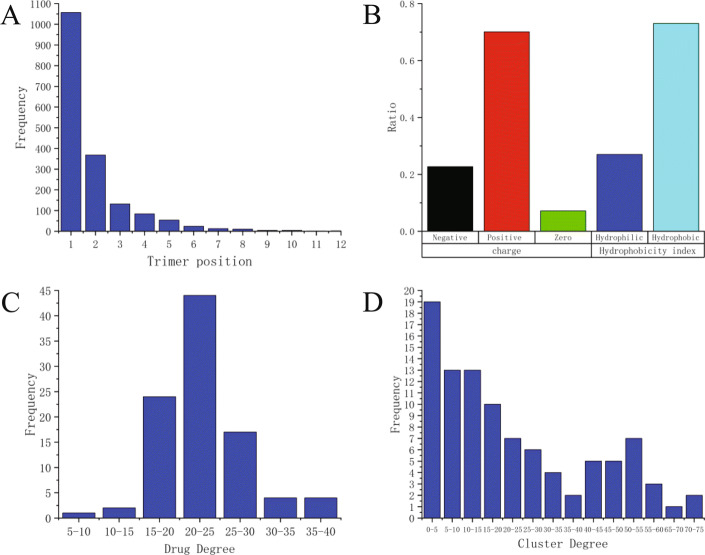
Investigation of the data set. **a** The distribution of trimmers. The abscissa shows the distribution of the trimer in DBPs and the ordinate shows the frequency. The results showed that 60.3% of the trimers are only located in the specific DBPs. **b** The nature of interaction data is charge and hydrophobicity analysis of trimmers. **c** The degree distributions of drugs. **d** The degree distribution of the clusters

We have investigated the DBP-drug interaction information in this work. The X-ray and other biology studies reveal that many proteins can have more than one drug binding site. For instance, some enzymes have two or more binding sites, one for inhibitor or activator and another for substrate. So, we constructed a binding site clusters-drug association network using a bipartite graph to check the degree distributions of both binding sites and drugs. We find that there is a local overlap in these drug-binding sites. Therefore, we analyzed the trimers that describe local information of binding sites to check the extent of overlap of local binding sites in different proteins (Fig. 2a). From Fig. 2a we can see that 39.7% of the trimers are located at the drug binding site of more than one protein, which is consistent with the fact that the drug binding sites of the proteins are partially overlapping.

The hydrophobicity and charge intensity of protein binding sites play an important role in the drug-protein binding process. Therefore, we analyze the hydrophobicity and charge intensity of the trimers. The results indicate that the drug tends to act on hydrophobic trimers and positively charged trimers (Fig. 2b). Normally, protein surface is surrounded by hydrophilic residues, and the residues with hydrophobic side chains are in principle located inside the molecule. This suggests that it is more likely to occur inside the protein during DBP-drug binding. The different charges are attracted to each other according to the electric field theory. This indicates that DBP is easier to bind to negatively charged drug molecules.

The degree distribution of the network reflects the sparsity of drug-cluster connections. Therefore, we analyze the network to check the degree distributions of both clusters and drugs (Fig. 2c and d). From Fig. 2c we can see that more than 87.6% drugs interact with clusters between 15 and 30 species. Figure 2d shows that more than 56.7% trimer clusters interact with less than 20 drugs. In all, we can infer that the connections of cluster-drug bipartite graph are sparse.

### Performance on the dataset

**Fig. 3:**
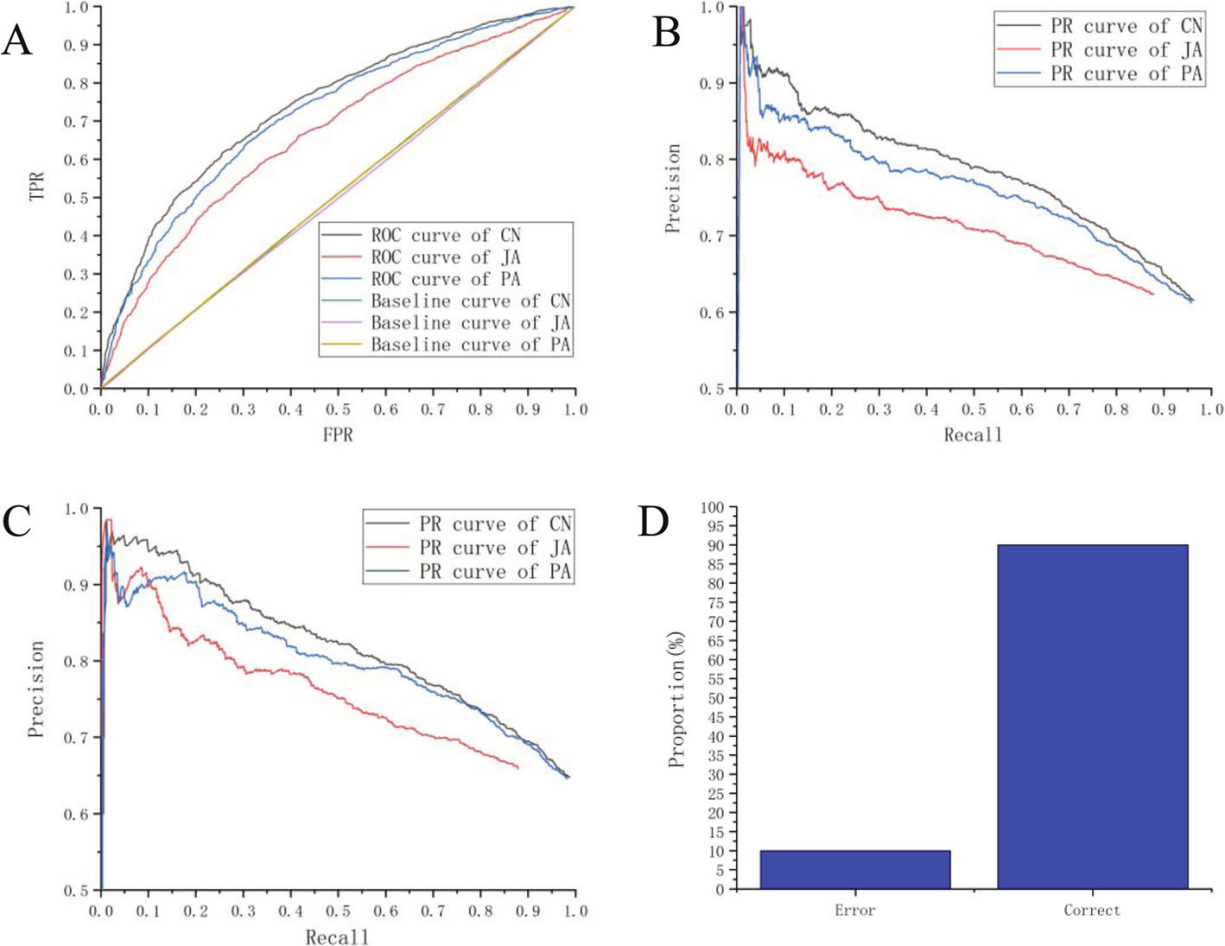
Model evaluation. **a** The ROC curves of drug-cluster. **b** The PR curves of drug-cluster. **c** The PR curves of drug-trimer. **d** The predicting accuracy on independent dataset

In order to make a fair comparison of these methods, the benchmark experiment was performed for each method. Benchmark experiments are randomly creating invalid interactions to mislead the performance of the method [22]. The model prediction performance can be adjusted by subtracting the prediction accuracy of the randomly generated interaction from the prediction accuracy of the known interaction. The experiment was repeated 100 times to obtain a baseline ROC curve on average. The results are shown in Fig. 3a. The baseline AUC for the three methods was 0.504, 0.498 and 0.504, respectively. This suggests that the prediction of random interactions by the three methods is consistent with random guessing. We can see that three methods have significantly higher precision than baseline. This shows that all three methods can make appropriate predictions. We compared the predictive ability of CN with JA and PA. In Fig. 3a, the ROC curve and the area under the curve (AUC) gained by various methods are shown. The AUC value obtained by the CN method was 0.732, which was significantly higher than the value of AUC obtained by using the JA (0.662) and PA (0.712) methods respectively. In Fig. 3b, the CN method is also significantly higher PR value than the other two methods (JA and PA). The above results show that the CN method has better predictive power than the PA and JA methods. Next, we measure the performance of the model based on the prediction of the DBP Trimer-Drug interaction. Figure 3c shows the PR curve using DBP Trimer-Drug as the dataset, and the results show that the performance of the CN method is also optimal. At the same time we measured the proportion of true/false DBPs in predictive models. As shown in Figure S1, more than 83.5% of DBPs are accurately recovered by the model. Finally, the independent dataset from the PDB database is used to verify the performance of the model. This independent dataset contains 20 DBP-drug complexes from PDB database (Table S1). The drug-trimers are extracted to test their accuracy. In Fig. 3d, the result shows that 90% of DBPs are correctly predicted by using the CN method.

In order to measure of the quality of these methods, we compared the AUC, AUPR, F1-score, Sensitivity and Specificity of JA, PA and the CN method respectively. As shown in Table 1, the CN method achieved better performance than other methods. The results show that the CN method has better predictive power than the PA and JA methods.

**Table 1:** The quantitative evaluations of the methods performance

Method	AUC	AUPR	F1-score	Sensitivity	Specificity
CN	0.732	0.755	0.751	0.963	0.602
JA	0.662	0.633	0.729	0.878	0.531
PA	0.712	0.731	0.746	0.957	0.597

Through the above analysis of predictive performance, the CN method was used to predict drug-cluster associations in the network. The score calculated by the CN method is used as the basis for network prediction. The possibility of drug-cluster association is judged based on the score.

### Network prediction model

In this section, we first give a brief overview of drug-cluster association prediction matrix. Then we investigate the underlying chemical mechanisms of drug-cluster associations. Finally, the DBP-drug interactions in the network prediction model are analyzed.

**Fig. 4:**
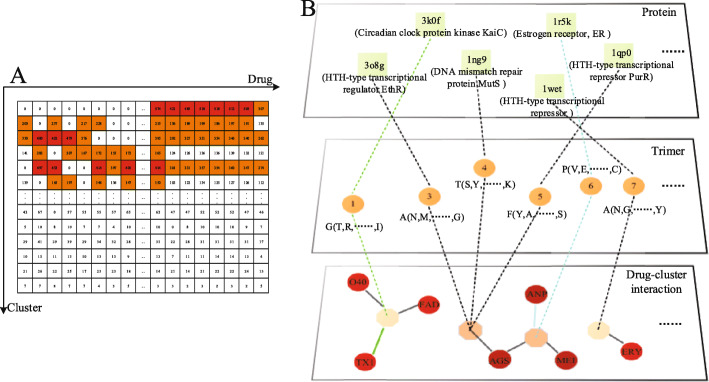
Interaction network analysis. **a** The predicted score matrix. The orange position is the significant interactions and the red position is the important interaction. **b** Drug-trimer-protein network prediction model, the first letter of trimer is the center amino acid of the trimer cluster, and the letters in the parenthesis represent the subordinate amino acids

Based on comparison with three methods, the CN method is used for link prediction. A prediction matrix of drug-cluster associations was constructed based on the scores calculated by the CN method (Fig. 4a). Although there are 7235 non-zero elements in the matrix, only those whose value is larger than 143 are viewed as significant (the average standard error is 143). As a result, there are 2822 significant interactions in the network. During the significant interactions, the interaction values larger than 404 (top 20%) are regarded as import.

**Fig. 5:**
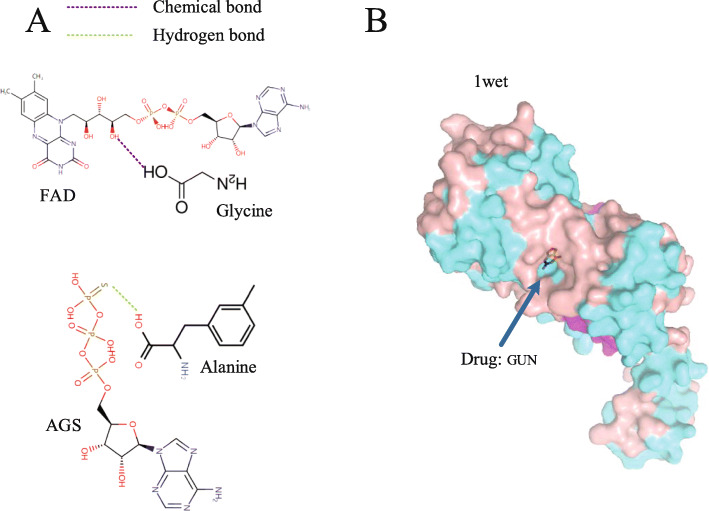
Analysis of prediction results. **a** The binding mechanism of the drug-trimers (amino acids). The binding of the drug-trimers (amino acid) is mainly the binding of chemical bonds and hydrogen bonds. **b** Complex of drug GUN and protein nuclear receptor coactivator 1 (PDB ID: 1wet)

Network visualization of predicted drug-trimer-DBPs could provide helpful information for research of intrinsic binding mechanisms and discovery of new therapeutic indications (Fig. 4b). According to the hypothesis, drug-clusters associations reflects chemical interaction, as a result, it is necessary to investigate whether the drug-clusters associations response the hypothesis. Since the clusters are composed of trimers, we investigate the hypothesis through drug-trimers interactions. For example, FAD (Flavin adenine dinucleotide, containing hydroxyl) and Glycine (containing carboxyl) react chemically to form lipids and water. In some situations, the major amino acid could not form significant chemical interaction with drug. However, if the distance and orientation are appropriate, the major amino acid can interact with the drug through hydrogen bonding. For example, a hydrogen atom in alanine is covalently bonded to an atom N having a large electronegativity. When the atomic S with large electronegativity and small radius in AGS (Adenosine 5’-[ *γ*-thio]triphosphate) is close, hydrogen is used as a medium between N and S to form a hydrogen bond. We only analyze the two interactions (Fig. 5), and the others could be analyzed similarly.

Next, we analyze the DBP-drug interactions in the network prediction model through the trimer as a link. In the drug-trimers-protein network prediction model (Fig. 5b), Erythromycin (ERY) can establish an interaction relationship with HTH-type transcriptional repressor (1wet) via trimer 7. HTH-type transcriptional repressor controls the expression of TtgABC efflux pump may be a factor in the resistance of bacteria [23]. Herein, HTH-type transcriptional repressor was predicted and verified as a new receptor for Erythromycin (ERY). Erythromycin is a bacteriostatic antibiotic widely used to treat various infections. The complex of GUN and HTH-type transcriptional repressor (1wet) showed that the binding process occurred inside nuclear receptor coactivator 1 (Fig. 5b), which verified that the binding process of the drug and DNA binding protein occurred inside the protein.

## Discussion

The PR curve and related indicators show that CN has the best performance among the 3 similarity indicators. This is beyond our expectations because CN is the simplest index. In general, more complex indexes should have better performance, because they have considered more information about the network structure. However, this is not the case with the drug-cluster association. This shows that the CN is actually a very powerful link prediction method even if it is very simple. The performance of PA is between CN and JA. PA is based on the Matthew effect, and the performance shows that the drug-cluster association may conform to the Matthew effect in social networks. JA shows the lowest performance out of all methods. Its poor performance may be because it solves the problem by placing more emphasis on the links of non-affected nodes to ensure that the common neighbors they share are due to their similarity rather than their impact.

## Conclusions

In this work, we consider binding is a local event and emphasize the local information in DBP-drug interaction prediction. We apply drug-clusters (The same type of trimer is included in the clusters) associations instead of DBP-drug interactions and propose a network prediction model to predict drug-trimers interactions and DBP-drug interactions. We first extracted the drug-binding sites from DBP-drug complexes. Then we broke the binding sites into trimers so that it was analyzed as local information, and clustered the same kind of trimers so that we can get the drug-clusters interaction. Finally, based on the link prediction, we proposed a new DBP-drug network prediction model.

Compared with traditional protein-drug networks, the proposed prediction network model has the advantage of finding trimer candidates and protein candidates simultaneously. Moreover, we no longer represent the protein as a whole but extract the drug-binding sites from DBP-drug complexes and apply the binding sites to describe protein information. Along this way, we can clearly know how the local binding site interacts with the drug. In all, we highlight the local binding site information of the protein during the binding process and attempt to figure out a clear relationship between the drug and the binding sites.

## Methods

### Materials

In this work, we extracted the DBP-drug complexes from scPDB database (http://bioinfo-pharma.u-strasbg.fr/scPDB/) [24], which is an annotated archive of the druggable binding sites extracted from the PDB database. Until 2019, we got the 4782 proteins and 6326 ligands from scPDB. After removing the redundancies and checking the consistency, we obtained 110 DBP-drug complexes, which include 97 drugs in this dataset (Table S2). Among them, 3463 single binding sites are extracted from protein-drug targets.

### The DBP-drug network model

**Fig. 6:**
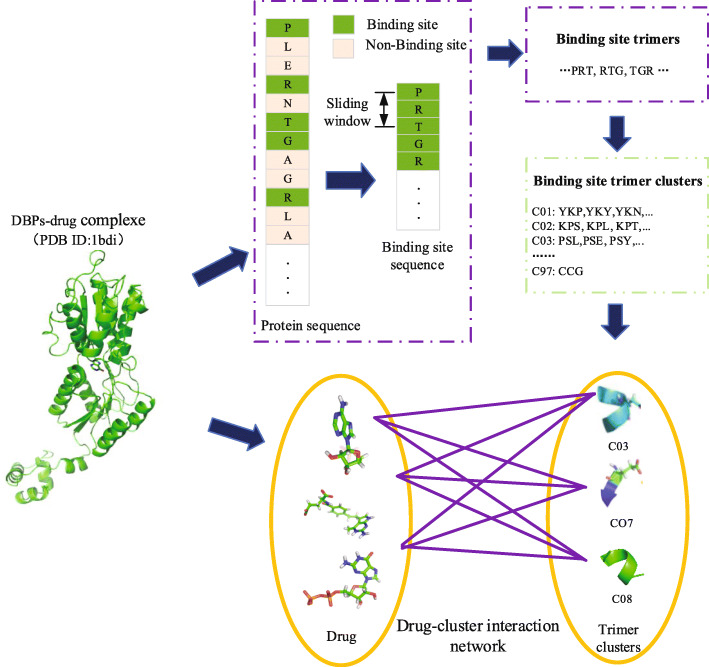
The flow chart is the drug-cluster associated model. The binding sites are first extracted from the PDB structure, and then we broke the binding site sequences into the trimers. All the trimmers are casted into 97 clusters (the type of drug). Because the trimers in the same cluster own similar chemical properties, the clusters can be viewed as chemica “group”. Each cluster in every binding site is used as groups-drug interaction pairs

The DBP-drug network will help to find some potential drug effects. The network is also a potential drug prediction model for DBPs. The prediction processes are broadly divided into the following sections. First, the drug binding sites of DBPs are extracted from the dataset, and the three consecutive sites are regarded as a binding unit. Then, the binding units are clustered based on hierarchical clustering algorithm according to their physical-chemical properties [25]. Because the units in the same cluster own similar physical-chemical properties, the clusters can be regarded as physical-chemical “groups”. The clustering groups are constructed for the link prediction with the drug interaction. The construction of the network model is shown in Fig. 6.

### Binding site trimer

The DBP-drug interaction is the binding of drug to local binding sites on the DBP sequence. Amino acid trimers are currently used to represent local binding sites [26]. In this work, we obtain binding sites of DBP-drug complexes from the scPDB database. Although these binding sites are discontinuous in sequence, they are relatively close in spatial structure. We connect these discontinuous amino acids to form a continuous sequence of binding sites. Then, the distance of 3 amino acids is used as the length of the window, and the sequence is slid to generate trimmers. For example, the binding site sequence NGMG generates two trimers NGM and GMG. The binding sites of DBPs are generated 3240 trimers (Table S3).

### Vector representation of trimers

The similar trimers are clustered together by the hierarchical clustering according to their physical-chemical properties [27]. After dividing the drug binding site into trimers, we treat each trimer as a chemical group to investigate the drug-trimer association. Then it is necessary to bring together trimers of the same nature. This way we can investigate the interaction between different trimers and drugs. Since these trimers are composed of natural amino acids, it is impossible to cluster them directly. Therefore we need to obtain the vector of trimers based on self-features.

The 237 physicochemical properties of amino acids were used to describe amino acids [28–30], such as residue volume, polarizability and solvation free energy (Table S4). An amino acid is represented as a 237-dimensional vector, resulting in a matrix X of 20 * 237 dimensions. And then, a principal component analysis (PCA) is applied to reduce the dimension. As a result, each amino acid is described by a 5-dimensional feature vector.

1 $$\begin{array}{@{}rcl@{}} X (*) = \left(\sqrt{\lambda_{1}}E_{1},\sqrt{\lambda_{2}}E_{2},\sqrt{\lambda_{3}}E_{3},\sqrt{\lambda_{4}}E_{4},\sqrt{\lambda_{5}}E_{5}\right). \end{array} $$

Where *X*(∗) represents a 5-dimensional vector of amino acid ∗, *E* represents an eigenvector, and *λ* represents an eigenvalue.

Amino acid trimers are represented by a single amino acid combination. The amino acid trimer is mapped into a 5-dimensional vector space, and the center (major) amino acid is highlighted his central role in each of trimer, and the right and left amino acid does not distinguish the order of the amino acids on both sides. The vector representation of the trimer as follows:

2 $$\begin{array}{@{}rcl@{}} \alpha_{tri} \left(\alpha_{01},\alpha_{00},\alpha_{10}\right) = X(\alpha_{00})+\frac{X\left(\alpha_{01}\right)+X\left(\alpha_{10}\right)}{4}. \end{array} $$

Where *α*_*tri*_ represents the five-dimensional vector of the trimer, *α*_00_ is the central (major) amino acid, and *α*_01_ and *α*_10_ are the left and right amino acids (subordinate) respectively.

### Trimers clustering

Trimers clustering owns similar physical-chemical properties, the clusters can be viewed as physical-chemical “group”. Here, since there is no need to specify the initial cluster center point, we use the hierarchical clustering method for clustering the trimers. The overall chemistry of the drug determines its efficacy. In this paper we consider a drug as a functional group. First the number of given clusters is 97, which is determined by the type of drug. These different drugs are composed of different chemical molecular structures, and the chemical nature of each drug is certain specific. As a drug is a chemical group that specifically binds to a class of physicochemical cluster groups, the drug-trimers are divided into 97 clusters to represent different functional groups, which will help to insight into the differences between different drug binding sites. Then, each trimer is treated as a separate class and the distance per 2 trimers is calculated. And then, the two trimers with the smallest distance are merged into the same class. Finally, the 3240 trimers are grouped under 97 clusters based on the 237 physical-chemical properties.

### Network description

Denoting the drug set as *D* = { *d*_1_, *d*_2_,..., *d*_*n*_} and cluster set as *C* = { *c*_1_, *c*_2_,..., *c*_*m*_}, the DCA (drug-cluster association) can be described as a bipartite DC graph *G*(*D*, *C*, *E*), where *E*(*e*_*ij*_: *d*_*i*_∈*D*, *c*_*j*_∈*C*). A link is drawn between *d*_*i*_ and *c*_*j*_ when the drug *d*_*i*_ is associated with the cluster *c*_*j*_. The DC bipartite network can be presented by an *n*×*m* adjacent matrix *a*_*ij*_, where *a*_*ij*_ = 1 if *d*_*i*_ and *c*_*j*_ is linked, otherwise *a*_*ij*_ = 0. The illustration of drug-cluster association network is shown in Figure S2.

### Network prediction method

Based on drug-cluster association, we applied link prediction methods to predict drug-cluster association. This method predicts based on the topology of the network and does not require additional feature information [31, 32]. Based on the successful experience of the relevant network, we selected the following three link prediction methods [17, 33]. The sample diagram of drug-cluster network is shown in Figure S3. In this paper, there are 2174 neighbors of drugs, and 51972 neighbors of clusters. The distribution of neighbors for drugs-clusters network is shown in Figure S4.

Common neighbor (CN) method: In the original CN index, *Γ*(*x*) and *Γ*(*y*) represent the set of neighbors of *x* and *y*, respectively. If two nodes *x* and *y* share many common neighbors, there may be a link between the two nodes.

3 $$\begin{array}{@{}rcl@{}} S_{xy}^{CN} = \left|\Gamma(x) \cap \Gamma(y)\right|. \end{array} $$

This essentially counts the number of nodes which have both *x* and *y* as their neighbor nodes. In a drug-cluster network, if we look at drug *x*, its neighbors will always be clusters. At the same time, there are no links between clusters and clusters. This means that the neighbor set of drugs and the neighbor set of clusters will not have an intersection. We can then redefine CN as:

4 $$\begin{array}{@{}rcl@{}} S_{xy}^{CN^{'}} = \left|\Gamma(x) \cap \hat{\Gamma}(y)\right|. \end{array} $$

Where $\hat {\Gamma }(y)$ is defined as the set of neighbors of cluster *y*^′^*s* neighbors, *Γ*(*x*) denote the set of neighbors of drug *x*.

Jaccard (JA) method: The JA index measures the probability that nodes *x* and *y* have common features. Taking into account the influence of a node in the network, the JA index is basically a normalized version of CN.

5 $$\begin{array}{@{}rcl@{}} S_{xy}^{Jaccard} = \frac{\left|\Gamma(x) \cap \Gamma(y)\right|}{\left|\Gamma(x) \cup \Gamma(y)\right|}. \end{array} $$

Similar to CN, we have to modify the JA index for drug-cluster binary network:

6 $$\begin{array}{@{}rcl@{}} S_{xy}^{Jaccard^{'}} = \frac{\left|\Gamma(x) \cap \hat{\Gamma}(y)\right|}{\left|\Gamma(x) \cup \hat{\Gamma}(y)\right|}. \end{array} $$

Where $\hat {\Gamma }(y)$ is defined as the set of neighbors of cluster *y*^′^*s* neighbors, *Γ*(*x*) denote the set of neighbors of drug *x*.

Preferential attachment (PA) method: PA means that nodes with multiple links tend to produce more new links.

7 $$\begin{array}{@{}rcl@{}} S_{xy}^{PA} = k_{x} \times k_{y}. \end{array} $$

Where *k*_*x*_ is the degree of drug *x*, *k*_*y*_ is the degree of cluster *y*.

### Evaluation method

In our experiments, we use 10-fold cross-validation, which is usually the preferential method in terms of bias and variance compared to regular cross-validation [34]. The dataset is randomly divided into the 10 non-overlapping subsets of the equal size in terms of the number. The model randomly select a subset, and the matched numbers of random sampled non-interacting pairs as test sets (There are 434 pairs in test sets, include 217 known interaction pairs and 217 non-interaction pairs). The remaining nine subsets are used to build the network model. This process is repeated ten times. The overall performance was calculated by averaging the performance of the 10 subsets (at the fold level). The true positive rate (*TPR*) and the false positive rate (*FPR*) are calculated the average from each iteration. During the prediction process, the score for the drug-cluster association is calculated. Then the score is used as a threshold. When the score is greater than or equal to the threshold, the predicted result is that there is an interaction, otherwise there is no interaction.

The false positive rate (*FPR*) is defined as:

8 $$\begin{array}{@{}rcl@{}} FPR = \frac{FP}{TN+FP}. \end{array} $$

The true positive rate (*TPR*) is defined as:

9 $$\begin{array}{@{}rcl@{}} TPR = \frac{TP}{TP+FN}. \end{array} $$

In our case of prediction, True positive (*TP*) refers to correctly predict the interaction. False positive (*FP*) refers to no interaction, but interaction is predicted. False negative (*FN*) refers to interactions, but predicts that there is no interaction. True negative (*TN*) refers to the correctly predicted that there is no interaction.

## Supplementary information

**Additional file 1** Figure S1 shows the proportion of true/false DBP that our model recover. Figure S2 Illustration of drug-cluster association network. A). Drug-protein complex. B). Trimers of binding sites as a unit. C). The trimers are clustered based on the physicochemical properties. D). Drug-cluster association network. E). Predicting drug target in the associated network. Figure S3 shows a sample diagram of drug-cluster networks. Figure S4 shows the distribution of neighbors for drugs-clusters network.

## Data Availability

The source and data are freely available at: https://github.com/HNUBioinformatics/Data-and-materials. Table S1: 20 DNA-binding protein-drug complexes (Test Set). Table S2: 110 DNA-binding protein-drug complexes (Data Set). Table S3: The binding sites of DBPs are generated 3240 trimers. Table S4: The 237 physicochemical properties of amino acids.

## Abbreviations

DBP: DNA binding protein
DCA: drug-cluster association
CN: common neighbor
NBI: network-based inference
PA: preferential attachment
JA: Jaccard
TPR: true positive rate
FPR: false positive rate
TP: true positive
FP: false positive
FN: false negative
TN: true negative
