# Supplementary material for: Predicting DNA binding protein-drug interactions based on network similarity
Source: BMC Bioinformatics. 2020 Jul 20;21:322. doi: 10.1186/s12859-020-03664-6 (PMC7372772; doi:10.1186/s12859-020-03664-6)
Supplement: Supplementary file 1 — Additional file 1 Figure S1 shows the proportion of true/false DBP that our model recover. Figure S2 Illustration of drug-cluster association network. A). Drug-protein complex. B). Trimers of binding sites as a unit. C). The trimers are clustered based on the physicochemical properties. D). Drug-cluster association network. E). Predicting drug target in the associated network. Figure S3 shows a sample diagram of drug-cluster networks. Figure S4 shows the distribution of neighbors for drugs-clusters network. [file 12859_2020_3664_MOESM1_ESM.docx]

**I.** The proportion of true/false DBP**.**

**
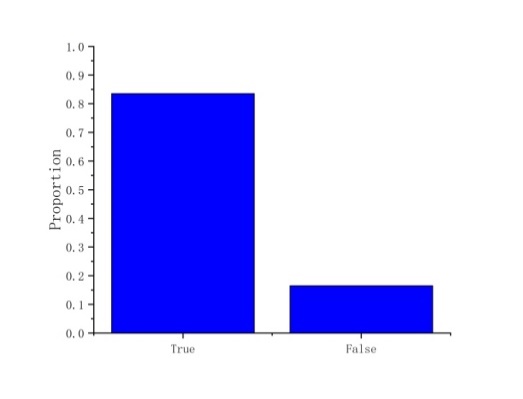
**

Figure S1. The proportion of true/false DBP.

**II. Illustration of Drug-cluster association network.**

**Figure S2 Illustration of Drug-cluster association network.** A). Drug-protein complex. B). Trimers of binding sites as a unit C). The trimers are clustered based on the physicochemical properties. D). Drug-cluster association network. E). Predicting drug target in the associated network.

**III. The sample diagram of Drug-Cluster networks.**

**Figure S3. The sample diagram of Drug-Cluster networks.**

**IV.** The distribution of neighbors for drugs-clusters network.

Figure S4. The distribution of neighbors for drugs-clusters network.
